# AngioPLUS-derived carotid intraplaque neovascularization predicts recurrent anterior circulation stroke: a prospective cohort study

**DOI:** 10.3389/fneur.2026.1819400

**Published:** 2026-08-11

**Authors:** Hao Zhang, Jun He, Jimei Xu, Rongfeng Wang, Shugang Cao, Mingwu Xia

**Affiliations:** 1 Department of Neurology, Hefei Hospital Affiliated to Anhui Medical University, The Second People's Hospital of Hefei, Hefei, China; 2 Department of Ultrasound, Hefei Hospital Affiliated to Anhui Medical University, The Second People's Hospital of Hefei, Hefei, China

**Keywords:** Intraplaque neovascularization, AngioPLUS technology, ischemic stroke recurrence, risk stratification, prospective cohort study

## Abstract

**Background and purpose:**

The predictive value of carotid intraplaque neovascularization (IPN), assessed by the novel AngioPLUS technique, for recurrent anterior circulation ischemic stroke (ACIS) remains unclear. This study aimed to evaluate the prognostic value of IPN for stroke recurrence in patients with ACIS and to develop a parsimonious prediction model by screening core predictors using Lasso regression, with subsequent assessment of its discrimination, calibration, and clinical utility.

**Methods:**

Consecutive patients admitted for ACIS who underwent IPN assessment using AngioPLUS ultrasound between August 2020 and February 2025 were prospectively enrolled. Independent predictors of recurrent ischemic stroke were identified using Cox proportional hazards regression models. Least absolute shrinkage and selection operator (Lasso) regression was employed to screen core predictors for constructing a parsimonious Cox model. Model discrimination was evaluated using time-dependent receiver operating characteristic (ROC) curves, and internal validation was performed with Bootstrap resampling B=1000 to calculate the concordance index (C-index). Model calibration was assessed using calibration plots. Risk stratification was conducted based on the optimal cut-off value of the total nomogram score, and differences in survival between groups were compared using the Kaplan–Meier method and the log-rank test.

**Results:**

A total of 143 patients (93 males, 65.0%; mean age 67.6 ± 11.7 years) with a median follow-up of 21 months (range 6–56 months) were enrolled, during which 26 patients experienced recurrent ischemic stroke. The recurrence rate was significantly higher in the high IPN group compared to the low IPN group (35.0% vs. 6.0%, *p* < 0.001). Lasso regression identified two core predictors: IPN Grades 2 (HR = 5.30, 95% CI 1.99–14.08, *p* < 0.001) and LDL-C (HR = 2.23, 95% CI 1.41–3.52, *p* < 0.001). The parsimonious model yielded a concordance index (C-index) of 0.786 (95% CI 0.707–0.866), with time-dependent AUC values of 0.716, 0.809, and 0.815 at 12, 24, and 36 months, respectively. Calibration plots closely approximated the ideal diagonal line. Nomogram-based risk stratification demonstrated that patients in the high-risk group had significantly lower recurrence-free survival rates at 12, 24, and 36 months (80.46, 36.40, and 28.31%, respectively) compared to those in the low-risk group (97.73, 90.80, and 88.21%, respectively). The median survival time was 23.0 months in the high-risk group and was not reached in the low-risk group (log-rank *p* < 0.001).

**Conclusion:**

Carotid IPN is an independent predictor of ACIS recurrence. A parsimonious Cox model incorporating IPN Grades 2 and LDL-C demonstrated favorable discrimination and calibration. The nomogram based on this model effectively identifies individuals at high risk of recurrence, offering a simple and practical tool for guiding individualized secondary prevention strategies in clinical practice.

## 1 Introduction

The carotid artery serves as a sentinel of systemic arterial health, and plaque vulnerability represents a cornerstone in the pathogenesis of ischemic stroke (1). Compared with stable plaques, vulnerable plaques confer a substantially higher risk of cerebral ischemia. Intraplaque neovascularization (IPN) is central to this vulnerability, as it promotes plaque progression and instability (2). By increasing the risk of intraplaque hemorrhage and subsequent rupture or embolization, IPN critically mediates the transition from asymptomatic atherosclerosis to acute clinical events (2, 3).

Although intravascular modalities such as optical coherence tomography (OCT) and intravascular ultrasound (IVUS) provide unparalleled microanatomical detail, their invasive nature precludes serial assessment in carotid artery disease (4, 5). Consequently, non-invasive surrogate markers are needed. Contrast-enhanced ultrasound (CEUS) has partially bridged this gap by linking IPN burden to stroke risk (6); however, its utility is limited by high cost, invasiveness, and contraindications to contrast agents (7, 8). AngioPLUS, a novel non-invasive microvascular Doppler technique, overcomes these limitations by capturing slow-flow microvascular signals without exogenous contrast agents (8, 9). Functionally, AngioPLUS reflects IPN hemodynamic activity rather than pure morphology, showing high diagnostic concordance with CEUS (area under the curve, AUC 0.94) and a strong correlation with histopathological microvessel density (4, 10, 11). Thus, it offers a clinically pragmatic tool for assessing plaque vulnerability (11).

Although IPN has been established as an independent risk factor for incident stroke (12, 13), its incremental predictive value for stroke recurrence beyond traditional risk factors remains undefined (13, 14). Most previous studies have focused on primary prevention, leaving a gap in prognostic modeling for secondary prevention (15). To address this gap while minimizing therapeutic confounding, we specifically targeted patients with mild-to-moderate carotid stenosis (<70% according to the North American Symptomatic Carotid Endarterectomy Trial, NASCET). Patients with severe stenosis (≥70%) typically undergo revascularization (carotid endarterectomy or stenting), which alters the natural history and obscures the prognostic value of plaque biology. In contrast, patients with mild-to-moderate stenosis—managed primarily with medical therapy—represent a “treatment-naive” population with an unmet need for refined risk stratification beyond conventional factors (e.g., age, blood pressure, and lipids).

Accordingly, this prospective cohort study was designed to test two hypotheses: first, whether AngioPLUS-assessed IPN is an independent predictor of recurrent anterior circulation ischemic stroke (ACIS) after adjustment for traditional risk factors; and second, whether a parsimonious prediction model integrating IPN and core clinical indicators can effectively stratify recurrence risk in this population.

## 2 Patients and methods

### 2.1 Study population

Between August 2020 and February 2025, a total of 170 consecutive patients with acute anterior circulation ischemic stroke (ACIS) and carotid atherosclerotic plaques were enrolled at the Second People’s Hospital of Hefei. The study protocol was approved by the Institutional Ethics Committee of the Second People’s Hospital of Hefei, and written informed consent was obtained from all participants or their legal representatives.

Inclusion criteria were as follows: (1) acute ischemic stroke confirmed by neuroimaging, with at least one ipsilateral carotid plaque (thickness >1.5 mm); and (2) mild-to-moderate carotid artery stenosis (<70%) according to the North American Symptomatic Carotid Endarterectomy Trial (NASCET) criteria (14).

All patients underwent etiological classification using the Chinese Ischemic Stroke Subclassification (CISS) system. Two neurologists independently assessed infarct distribution patterns, identified the culprit vessel, and evaluated plaque characteristics. After systematically ruling out cardioembolism, small artery occlusion, and other etiologies, the stroke was attributed to ipsilateral carotid large-artery atherosclerosis.

Exclusion criteria were as follows: (1) extensive plaque calcification precluding reliable assessment of intraplaque neovascularization (IPN); (2) total carotid artery occlusion or a history of carotid endarterectomy or stenting; (3) stroke etiologies other than large-artery atherosclerosis (e.g., cardioembolism, small-vessel occlusion, or undetermined etiology according to the TOAST criteria); (4) isolated posterior circulation infarction; (5) concurrent intracerebral hemorrhage or massive cerebral infarction; (6) severe systemic comorbidities, including cardiopulmonary dysfunction, hepatic or renal failure, or malignancy; (7) concurrent infection or autoimmune disease; and (8) incomplete clinical or imaging data.

### 2.2 Collection of clinical data

Comprehensive baseline data, including demographic characteristics (sex and age), lifestyle factors (smoking and alcohol consumption), and cerebrovascular risk factors (hypertension, diabetes mellitus, and hyperlipidemia), were collected for all patients. Medical history, encompassing coronary heart disease, prior ischemic stroke, and the use of pharmacological treatments (anticoagulants, antiplatelet agents, lipid-lowering agents, antidiabetic drugs, and antihypertensive agents), was also recorded. Systolic blood pressure (SBP) and diastolic blood pressure (DBP) were recorded upon admission.

Post-discharge medication adherence was evaluated by assessing compliance with prescribed oral regimens, which included antiplatelet agents, antidiabetic drugs, antihypertensive agents, and lipid-lowering agents. Reasons for treatment discontinuation were investigated, and patients who discontinued any prescribed medication for more than 1 month were classified as having poor medication adherence (16).

### 2.3 Laboratory parameters

Fasting venous blood samples were collected from all participants in the early morning following an overnight fast. Standard laboratory evaluations included complete blood counts, biochemical analyses, and coagulation profiling. Complete blood counts were determined using the Sysmex XN-10 automated hematology analyzer (Sysmex Corp., Kobe, Japan). Concentrations of fasting blood glucose (FBG), glycated hemoglobin (HbA1c), triglycerides (TG), total cholesterol (TC), high-density lipoprotein cholesterol (HDL-C), low-density lipoprotein cholesterol (LDL-C), homocysteine (Hcy), and creatinine (Cr) were measured using an automated biochemical analyzer (Hitachi 7,600–020; Hitachi High-Tech, Tokyo, Japan).

### 2.4 Diagnosis of carotid plaque and IPN evaluation

Conventional carotid ultrasonography was performed with patients in the supine position and the neck fully exposed. Carotid intima-media thickness (CIMT) was initially measured along the course of the artery. The common carotid artery, carotid bifurcation, and internal and external carotid arteries were examined for the presence of plaques, and detailed records were maintained regarding plaque location, size (maximum thickness and length), number, and echogenicity. Plaque echogenicity was graded according to the Gray-Weale visual classification system as follows: uniformly hypoechoic (Type I), predominantly hypoechoic (Type II), predominantly hyperechoic (Type III), uniformly hyperechoic (Type IV), or extensively calcified (Type V) (17). Carotid stenosis was categorized as mild (< 50%), moderate (50–69%), or severe (≥70%) based on the North American Symptomatic Carotid Endarterectomy Trial (NASCET) criteria (14).

Intraplaque neovascularization (IPN) in the target plaque was then evaluated using the SuperSonic Imagine AixPlorer system (Sonic Red Series; SuperSonic Imagine, Aix-en-Provence, France), equipped with an SL10-2 probe and integrated AngioPLUS microvascular imaging technology. The morphology (e.g., punctate or linear distribution), location, and flow spectral characteristics of the IPN signals were recorded.

**Figure 1:**
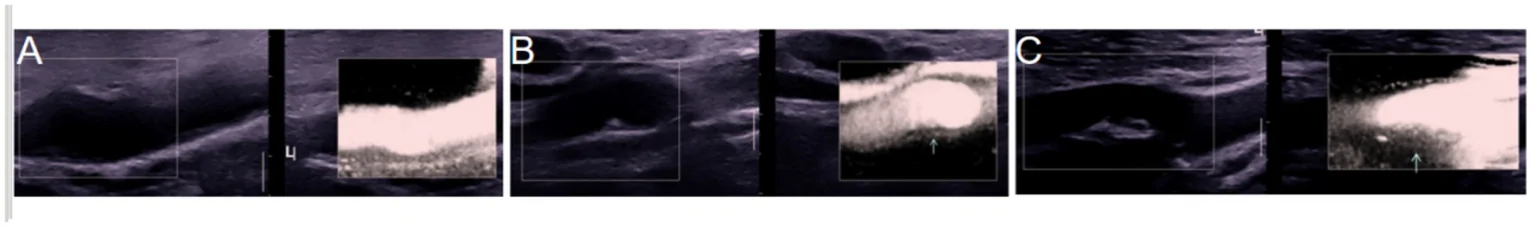
Carotid IPN grading. **(A)** Grade 0 IPN; **(B)** Grade 1 IPN; **(C)** Grade 2 IPN. In each panel, the left portion displays the two-dimensional B-mode ultrasound image, and the right portion shows the corresponding AngioPLUS imaging visualizing intraplaque microvascular flow signals.

Under AngioPLUS mode, IPN was identified by the presence of short-linear or strip-like high-signal flow echoes captured within a 2-min dynamic clip, and was graded as follows: Grade 0, no detectable flow signals within the plaque; Grade 1, sparse punctate or short-linear flow signals (< 4 signals) located unilaterally within the plaque; and Grade 2, dendritic or diffuse linear flow signals within the plaque. For clinical analysis, IPN grades were categorized into low IPN (Grades 0–1) and high IPN (Grade 2) (Figure 1).

All ultrasound examinations were independently evaluated by two trained sonographers (with 5 and 8 years of vascular ultrasound experience, respectively) who were blinded to the patients’ clinical and laboratory data. Prior to the formal study, both sonographers underwent a training session using 20 pilot images to standardize the grading criteria. In cases of disagreement, a third senior sonologist (with 15 years of experience) adjudicated the final grade. To assess inter-observer reliability, the weighted Kappa coefficient (with quadratic weights) was calculated for the ordinal three-tiered IPN grading (grades 0, 1, and 2), and the unweighted Kappa coefficient was calculated for the binary classification (low IPN vs. high IPN). The proportion of cases requiring adjudication by the third reviewer was also recorded.

### 2.5 Follow-up and endpoints

All enrolled patients with ACIS were followed up at 6-month intervals via telephone interview or outpatient clinic visit. When recurrent stroke was suspected, patients were promptly readmitted for diffusion-weighted imaging (DWI) to confirm acute ischemic infarction. Recurrent ischemic stroke was independently adjudicated by two neurologists not involved in patient enrollment, based on new neurological deficits and corresponding acute DWI lesions, in accordance with the same CISS classification and culprit vessel identification procedures used at baseline. Disagreements were resolved by a third senior neurologist. The primary endpoint was the incidence of ipsilateral ACIS recurrence confirmed by DWI. Follow-up was completed on August 30, 2025.

### 2.6 Statistical analysis

Statistical analyses were conducted using R software (version 4.5.0). A two-tailed *p* < 0.05 was considered statistically significant. Continuous variables with normal distribution were presented as mean ± standard deviation, whereas non-normally distributed variables were expressed as median (interquartile range). Intergroup comparisons were performed using the Student’s *t*-test, Mann–Whitney U test, or chi-square test, as appropriate.

Univariable Cox regression was used to identify potential predictors of stroke recurrence (*p* < 0.05). Variables meeting this threshold were entered into a multivariable Cox model to evaluate the independent association between IPN and ACIS recurrence. To reduce overfitting and select core predictors, least absolute shrinkage and selection operator (Lasso) regression was performed with *λ* = 0.106, after which a parsimonious Cox model was constructed.

Predictive performance was assessed using time-dependent receiver operating characteristic (ROC) curves, with the area under the curve (AUC) calculated at 12, 24, and 36 months. Discriminative ability and stability were evaluated by computing the time-dependent concordance index (C-index) with 95% confidence intervals using bootstrap resampling (1,000 replicates). A nomogram based on the parsimonious model was developed to predict 12-, 24-, and 36-month recurrence-free survival, and its calibration was assessed with calibration plots via bootstrap resampling (1,000 replicates). The optimal cut-off value for the total nomogram score was determined using the Youden index, and patients were stratified into low- and high-risk groups accordingly. Survival curves were generated using the Kaplan–Meier method, and differences between groups were compared with the log-rank test.

## 3 Results

### 3.1 Study population and baseline clinical characteristics

**Figure 2:**
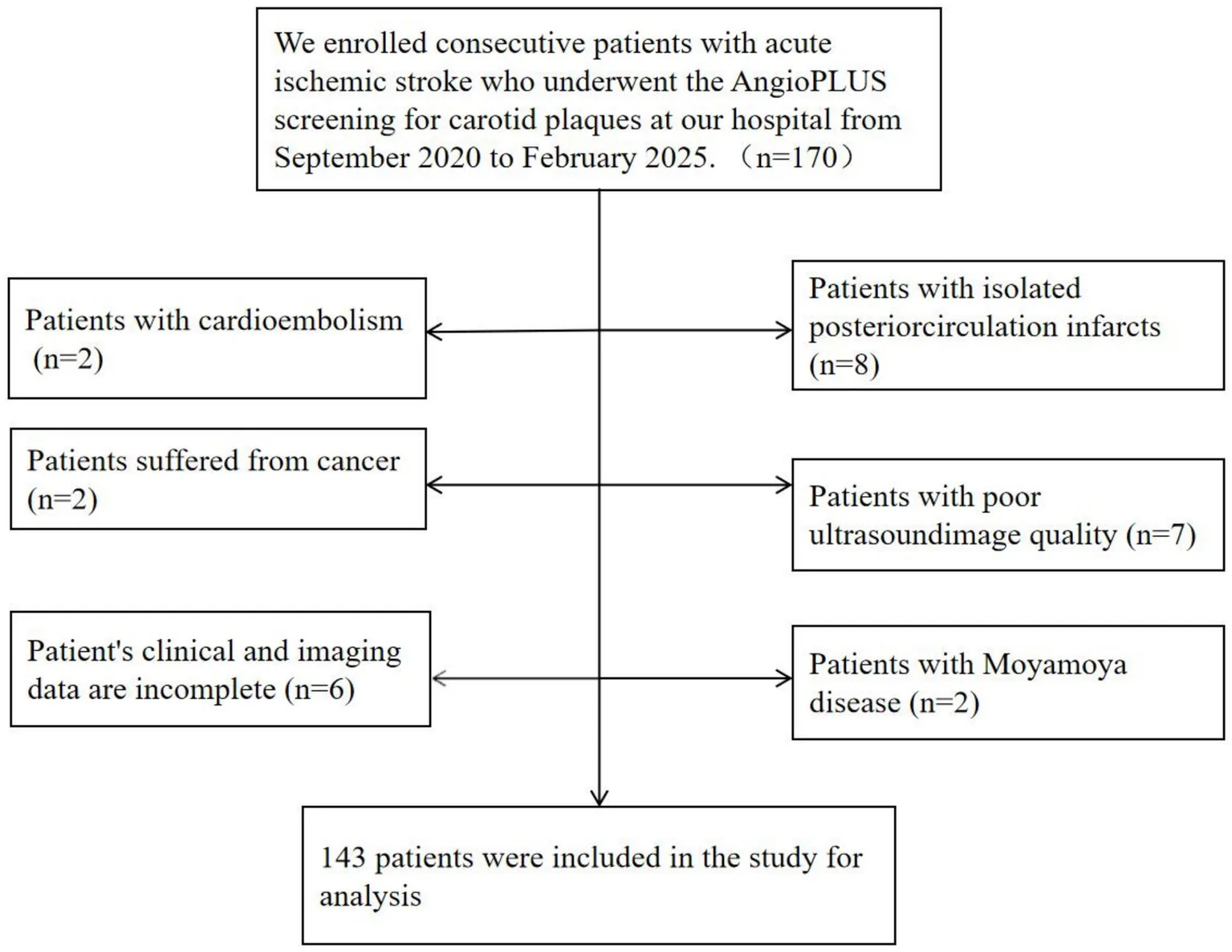
Study flowchart.

Initially, 170 patients with symptomatic carotid artery stenosis were screened for eligibility. Of these, 27 were excluded for the following reasons: poor-quality ultrasonographic images (*n* = 7), isolated posterior circulation infarction (*n* = 8), cardioembolism (*n* = 2), malignancy (*n* = 2), Moyamoya disease (*n* = 2), and incomplete clinical or imaging data (*n* = 6). Consequently, 143 patients (93 men, 50 women; mean age, 67.6 ± 11.7 years) were included in the final analysis (Figure 2). Patients were then stratified into a low IPN group (grades 0–1, *n* = 83) and a high IPN group (grade 2, *n* = 60). Baseline characteristics are summarized in Table 1. No significant differences were observed between the two groups in traditional risk factors, including age, sex, hypertension, diabetes mellitus, and hyperlipidemia, or in most laboratory parameters (all *p* > 0.05). Notably, the proportion of patients with a history of prior stroke was significantly higher in the high IPN group than in the low IPN group (36.7% vs. 18.1%, *p* = 0.012). Additionally, the recurrence rate was markedly higher in the high IPN group (35.0% vs. 6.0%, *p* < 0.001). During a median follow-up of 21 months (range, 6–56 months), recurrent ischemic stroke occurred in 26 patients: 5 (6.0%) in the low IPN group and 21 (35.0%) in the high IPN group. Baseline characteristics stratified by recurrence status are presented in Supplementary Table 1.

**Table 1:** Baseline characteristics of the study participants stratified by intraplaque neovascularization grade.

Variable	Low IPN (*n* = 83)	High IPN (*n* = 60)	*p*-value
Age, year, mean ± SD	66.31 ± 12.51	69.30 ± 10.46	0.134
Sex, man, *n* (%)	51(61.4)	41(68.3)	0.396
Hypertension, *n* (%)	42(50.6)	28(46.7)	0.642
Diabetes, *n* (%)	22(26.5)	15(25)	0.839
Hyperlipidemia, *n* (%)	20(24.1)	16(26.7)	0.727
Coronary heart disease, *n* (%)	11 (13.3)	9(15)	0.766
History of stroke, *n* (%)	15 (18. 1)	22(36.7)	0.012
Smoking, *n* (%)	25 (30.1)	24(40)	0.219
Drinking, *n* (%)	24 (28.9)	17(28.3)	0.939
Poor medication adherence, *n* (%)	21 (25.3)	9(15)	0.135
BMI, mean ± SD	24.46 ± 2.76	24.51 ± 2.53	0.914
TG, median (IQR)	1.46 (1.10, 2.36)	1.31(0.86, 1.95)	0.270
TC, median (IQR)	4.54(3.83, 5.14)	4.31(3.70, 4.91)	0.163
HDL-C, mean ± SD	1.22 ± 0.28	1.19 ± 0.27	0.475
LDL-C, mean ± SD	2.93 ± 0.82	2.83 ± 0.86	0.481
FBG, median (IQR)	5.29 (4.78, 8.19)	5.77 (5.03, 7.05)	0.980
HCY, median (IQR)	12.10 (10.20, 17.40)	13.25(10.53, 16.93)	0.374
Cr, median (IQR)	69.10(55.70, 82.80)	66.75(56.95, 80.15)	0.659
Thrombolysis, *n* (%)	10(12.0)	6(10)	0.701
CIMT	1.0(0.8, 1.1)	0.950 (0.81, 1.10)	0.906
Recurrent, *n* (%)	5(6)	21(35)	<0.001
Plaque echogenicity, *n* (%)			0.767
Types I and II, *n* (%)	59(71.1)	44(73.3)	
Types III and IV, *n* (%)	24(28.9)	16(26.7)	
Degree of carotid stenosis			0.820
Mild stenosis, *n* (%)	69(83.1)	49(81.7)	
Moderate stenosis, *n* (%)	14(16.9)	11(18.3)	
PSV, mean ± SD	114.69 ± 11.15	116.25 ± 10.90	0.405

BMI, body mass index; SBP, systolic blood pressure; DBP, diastolic blood pressure; TG, triglycerides; TC, total cholesterol; HDL-C, high-density lipoprotein cholesterol; LDL-C, low-density lipoprotein cholesterol; FBG, fasting blood glucose; HCY, homocysteine; Cr, creatinine; CIMT, carotid intima-media thickness; PSV, peak systolic velocity.

Patients were stratified into the recurrence (*n* = 26) and non-recurrence (*n* = 117) groups based on the incidence of recurrent ischemic stroke. Baseline characteristics are detailed in Supplementary Table 1. No significant intergroup differences were observed in demographic parameters. However, compared with the non-recurrence group, the recurrence group demonstrated significantly higher levels of fasting blood glucose (*p* = 0.012) and homocysteine (*p* = 0.018), as well as a higher prevalence of grade 2 IPN (*p* = 0.002).

Among all 143 patients, the inter-observer agreement rate for IPN grading was 89.5% (128/143), with a weighted Kappa coefficient of 0.86 (95% CI: 0.79–0.93), indicating excellent agreement. For the binary classification (low IPN vs. high IPN), the unweighted Kappa coefficient was 0.84 (95% CI: 0.76–0.92). A total of 15 cases (10.5%) required adjudication by a third reviewer; of these, 13 (86.7%) were resolved by adopting the third reviewer’s assessment, and the remaining 2 (13.3%) were resolved by consensus discussion. These findings indicate that IPN grading in this study demonstrated high reproducibility.

### 3.2 Association between IPN grading and the risk of recurrent acute ischemic stroke

Univariable Cox regression analysis identified diabetes mellitus (HR 3.274, 95% CI 1.466–7.312; *p* = 0.004), poor medication adherence (HR 2.399, 95% CI 1.034–5.567; *p* = 0.042), elevated LDL-C levels (HR 2.195, 95% CI 1.375–3.504; *p* < 0.001), and grade 2 IPN (vs. grade 0–1; HR 4.936, 95% CI 1.859–13.105; *p* = 0.001) as significant predictors of recurrent ischemic stroke. Variables with *p* < 0.05 in the univariable analysis were subsequently entered into a multivariable Cox regression model. After adjustment, grade 2 IPN remained an independent predictor of recurrence (HR 6.936, 95% CI 2.464–19.524; *p* < 0.001) (Table 2).

**Table 2:** Predictive factors for future ischemic stroke.

Variable	Univariable analysis			Multivariable analysis		
	HR	95%CI	*P*-value	HR	95%CI	*P*-value
Age	1.009	0.975 ~ 1.043	0.613	-	-	-
Male	1.293	0.562 ~ 2.975	0.546	-	-	-
Hypertension	1.408	0.651 ~ 3.046	0.385	-	-	-
Diabetes	3.274	1.466 ~ 7.312	0.004	2.253	0.967 ~ 5.247	0.060
Hyperlipidemia	1.149	0.430 ~ 3.071	0.782	-	-	-
Coronary heart disease	1.589	0.547 ~ 4.620	0.395	-	-	-
History of stroke	1.381	0.615 ~ 3.100	0.434	-	-	-
Smoking	1.386	0.628 ~ 3.060	0.419	-	-	-
Drinking	1.256	0.545 ~ 2.898	0.592	-	-	-
Poor medication adherence	2.399	1.034 ~ 5.567	0.042	3.385	1.319 ~ 8.685	0.011
BMI	1.149	0.968 ~ 1.364	0.112	-	-	-
SBP	0.991	0.975 ~ 1.008	0.287	-	-	-
DBP	1.001	0.977 ~ 1.026	0.932	-	-	-
TG	0.926	0.622 ~ 1.376	0.702	-	-	-
TC	1.155	0.763 ~ 1.750	0.495	-	-	-
HDL-C	0.752	0.167 ~ 3.389	0.711	-	-	-
LDL-C	2.195	1.375 ~ 3.504	<0.001	1.825	1.128 ~ 2.953	0.014
FBG	1.127	0.995 ~ 1.277	0.059	-	-	-
HCY	1.030	0.991 ~ 1.070	0.130	-	-	-
Cr	1.003	0.985 ~ 1.023	0.719	-	-	-
CIMT	1.093	0.120 ~ 9.947	0.937	-	-	-
Carotid IPN (Grade 0–1)	Ref	Ref	Ref			
Carotid IPN (Grade 2)	4.936	1.859 ~ 13.105	0.001	6.936	2.464 ~ 19.524	<0.001
Types III and IV	Ref	Ref	Ref			
Types I and II	1.136	0.493 ~ 2.615	0.765	-	-	-
Mild stenosis	Ref	Ref	Ref			
Moderate stenosis	1.626	0.681 ~ 3.878	0.273	-	-	-

MI, body mass index; CIMT, carotid intima-media thickness; Cr, creatinine; FBG, fasting blood glucose; Hcy, homocysteine; HDL-C, high-density lipoprotein cholesterol; IPN, intraplaque neovascularization; IQR, interquartile range; LDL-C, low-density lipoprotein cholesterol; TC, total cholesterol; TG, triglycerides.

### 3.3 Lasso regression analysis and development of a parsimonious Cox predictive model

Lasso regression was performed for variable selection. At the optimal penalty parameter *λ* (lambda.1se = 0.106), two variables with non-zero coefficients were identified: LDL-C (coefficient 0.1081) and IPN grade 2 (coefficient 0.2481) (Supplementary Figure 1). These variables were subsequently entered into a parsimonious Cox proportional hazards model. As shown in Table 2, both IPN grade 2 (HR 5.30, 95% CI 1.99–14.08; *p* < 0.001) and LDL-C (HR 2.23, 95% CI 1.41–3.52; *p* < 0.001) were independent predictors of stroke recurrence. The model demonstrated good discriminative ability, with a concordance index (C-index) of 0.786 (95% CI 0.707–0.866).

### 3.4 Assessment of model discrimination and calibration

#### 3.4.1 Discrimination

**Figure 3:**
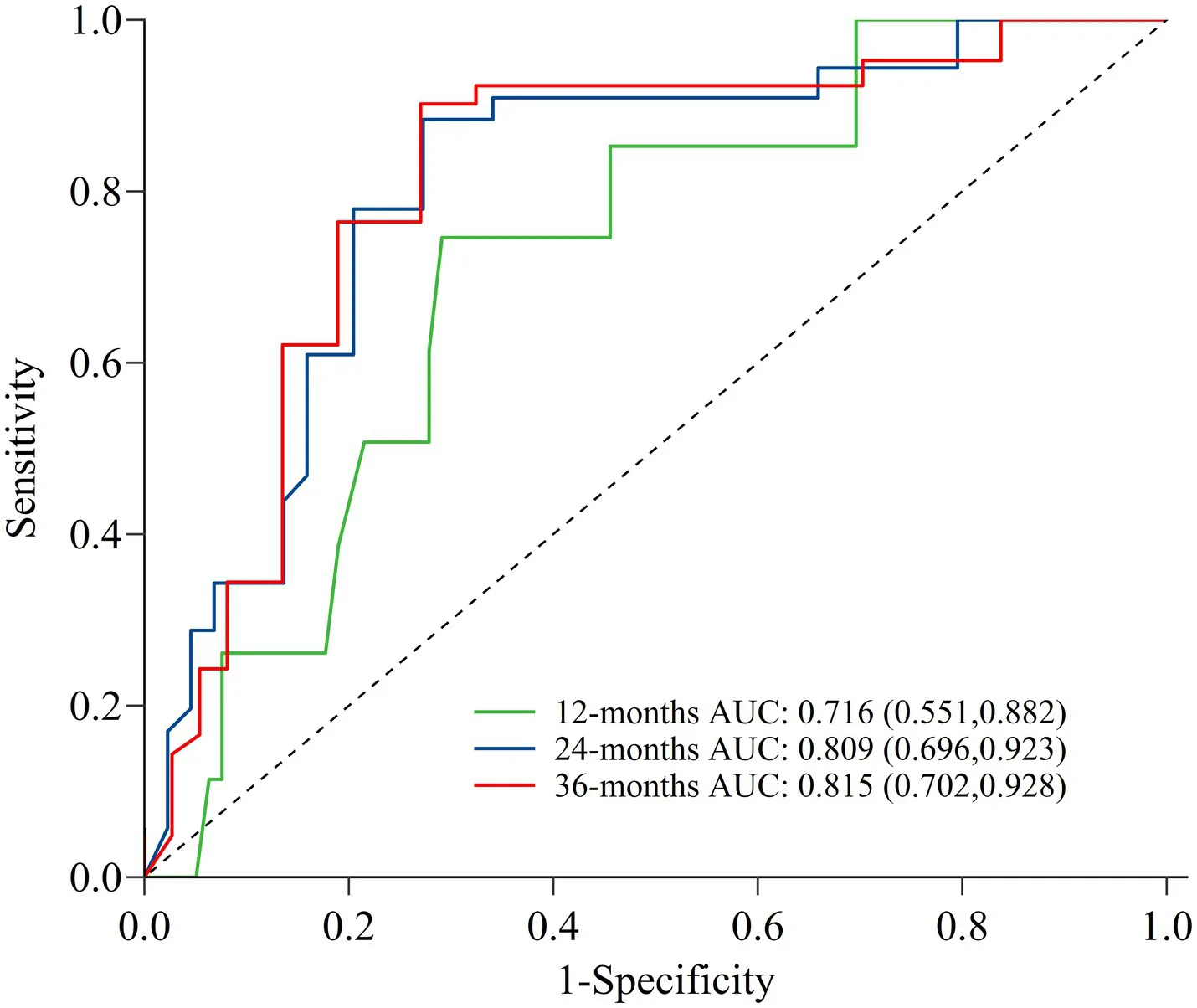
Time-dependent ROC curves of the parsimonious Cox model for predicting ACIS recurrence at 12, 24, and 36 months.

Time-dependent ROC curves yielded AUC values of 0.716 (95% CI 0.551–0.882), 0.809 (95% CI 0.696–0.923), and 0.815 (95% CI 0.702–0.928) at 12, 24, and 36 months, respectively (Figure 3). Bootstrap validation confirmed that the time-dependent C-index remained stable throughout follow-up, ranging from 0.71 to 0.90, remaining above 0.77 for most time points, and peaking at 0.90 at 54 months (Supplementary Figure 2). These findings indicate satisfactory discriminative ability of the model over time, with stable predictive performance.

### 3.5 Calibration

**Figure 4:**
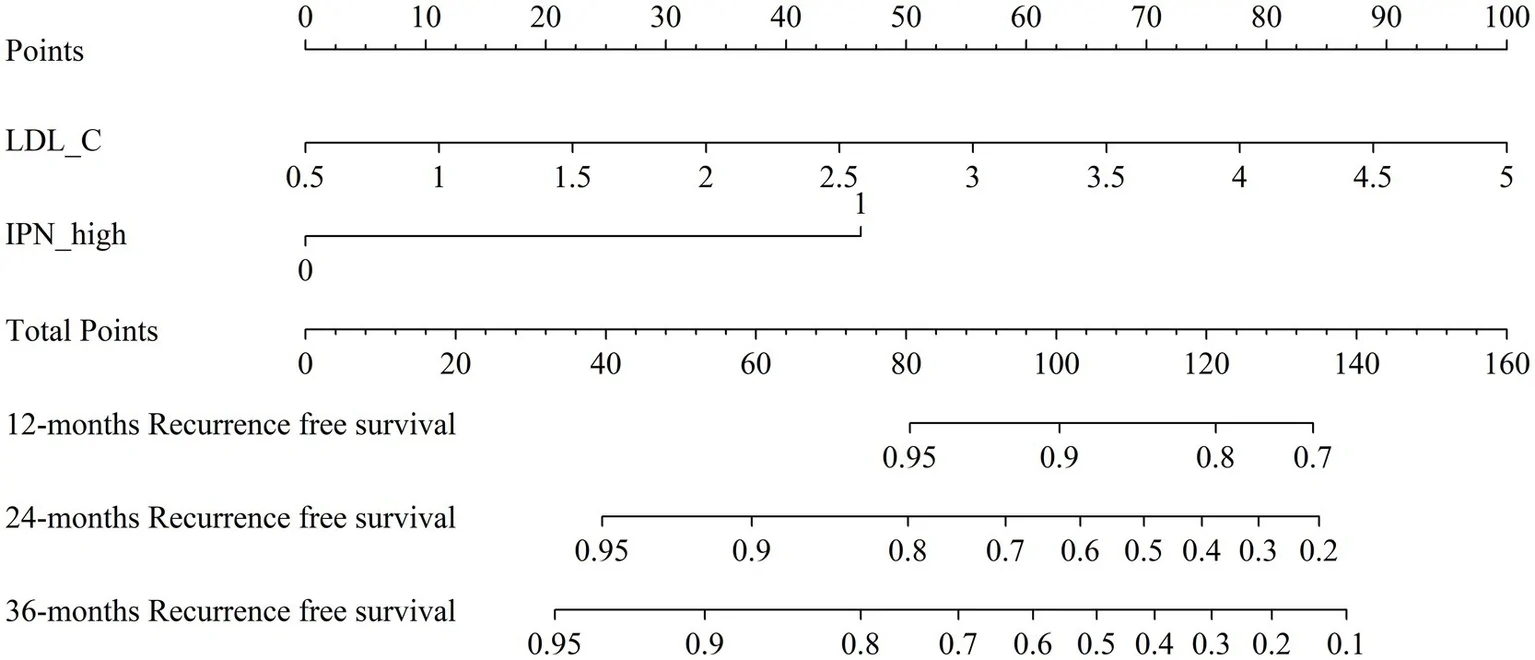
Nomogram for predicting 12-, 24-, and 36-month recurrence-free survival based on the parsimonious Cox model. To use the nomogram, locate the patient’s LDL-C value and IPN grade on each axis, draw a vertical line to the ‘Points’ axis to obtain the score for each variable, sum the scores, and locate the total score on the ‘Total Points’ axis. Draw a vertical line down to the survival axes to obtain the predicted recurrence-free survival probabilities.

A nomogram based on the parsimonious model was constructed to predict recurrence-free survival probabilities at 12, 24, and 36 months for individual patients (Figure 4). Model calibration was assessed using bootstrap resampling (1,000 replicates), and the bias-corrected calibration curves closely approximated the ideal diagonal line, indicating good calibration and model robustness (Supplementary Figure 3).

### 3.6 Risk stratification and survival analysis

**Figure 5:**
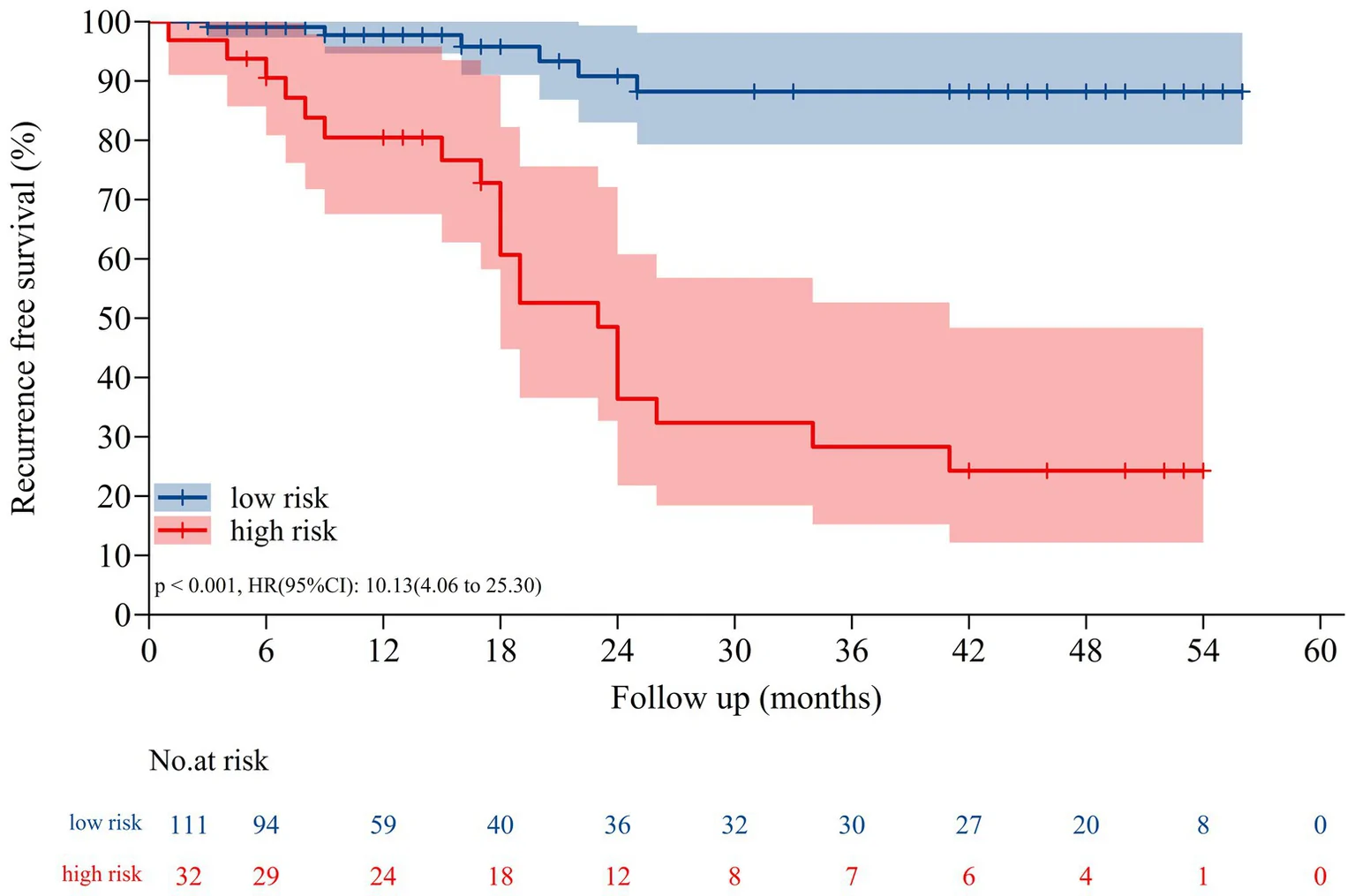
Kaplan–Meier survival curves for recurrence-free survival stratified by nomogram-derived risk groups. Patients were divided into low-risk (total score < 101.10) and high-risk (total score ≥ 101.10) groups based on the optimal cutoff value. The shaded areas represent 95% confidence intervals.

Patients were stratified into low-risk (total score < cut-off) and high-risk (total score ≥ cut-off) groups based on the nomogram-derived cut-off value of 101.10 points. Kaplan–Meier analysis showed that recurrence-free survival declined more slowly in the low-risk group, with rates of 97.73, 90.80, and 88.21% at 12, 24, and 36 months, respectively. In contrast, the high-risk group exhibited a rapid decline, with corresponding rates of 80.46, 36.40, and 28.31% at the same time points. The survival curves separated early during follow-up and continued to diverge progressively over time. Median survival was not reached (NR) in the low-risk group, whereas it was 23.0 months (95% CI 18.0–41.0) in the high-risk group (Figure 5). The log-rank test confirmed a significant difference in recurrence-free survival between the two groups (*p* < 0.001), demonstrating robust prognostic discrimination of the nomogram-based risk stratification model.

## 4 Discussion

This study identifies carotid intraplaque neovascularization (IPN), visualized by AngioPLUS, as an independent predictor of ipsilateral acute ischemic stroke (ACIS) recurrence. Lasso-penalized selection integrated with a parsimonious Cox model identified IPN grade 2 and LDL-C as the two strongest predictors, yielding a C-index of 0.786 and stable time-dependent AUCs (0.716–0.815). The nomogram derived from this two-variable model stratified patients into prognostically distinct groups (median survival: 23.0 months for high-risk vs. not reached for low-risk; log-rank test, *p* < 0.001). These results suggest that this simplified, imaging-plus-laboratory tool could be embedded into outpatient workflows to guide the intensity of secondary prevention—for example, by triggering more frequent surveillance or earlier lipid-lowering intensification in high-risk individuals.

Carotid IPN is a well-established marker of plaque vulnerability; however, its translation into longitudinal risk prediction models has remained limited. Although Cheng et al. linked IPN to adverse 90-day functional outcomes after ischemic stroke, their study was restricted to short-term prognosis and did not address recurrent vascular events (18). Although Zhang et al. demonstrated that IPN independently predicted future ischemic events in patients with mild carotid stenosis, their cohort was heterogeneous, comprising both symptomatic and asymptomatic individuals, and did not focus specifically on recurrence after an index stroke (19). Although Cui et al. proposed combining IPN with a baseline clinical model to improve risk identification, their analysis relied on conventional regression without formal variable selection or penalization (20).

The present study extends these observations by constructing a parsimonious, Lasso-regularized prediction model specifically for recurrent ipsilateral ACIS. The resulting two-variable model offers a simpler alternative for clinical use while maintaining strong discrimination and calibration, thereby addressing the translational gap between single-marker association studies and clinically deployable risk tools.

The co-selection of IPN grade 2 and LDL-C reflects a biologically coherent axis. LDL-C drives lipid deposition and, via oxidized LDL, upregulates VEGF-mediated angiogenesis, directly promoting IPN formation (21). In this cohort, LDL-C concentrations were significantly elevated in the high-IPN group (*p* < 0.05), and experimental evidence indicates that each 1 mmol/L increase in LDL-C raises IPN detection risk by 37%, while IPN progression reciprocally accelerates lipid core expansion and fibrous cap thinning. These data suggest that the IPN–LDL-C interplay identifies a subset of patients in whom aggressive lipid lowering may simultaneously target two synergistic recurrence mechanisms, providing a rationale for incorporating plaque imaging into treat-to-target lipid strategies.

The metabolic milieu underlying IPN further reinforces this concept. Clustering of diabetes, elevated fasting glucose, homocysteine, and LDL-C was more pronounced in the high-IPN group, consistent with prior work linking hyperglycemia, non-HDL-C, and obesity indices to IPN severity (15, 19, 21–23). An elevated neutrophil-to-lymphocyte ratio has also been associated with grade 2 IPN, emphasizing the contribution of systemic inflammation (24). These observations suggest that IPN grading may serve as an integrative imaging readout of the metabolic-inflammatory burden driving plaque progression, enabling clinicians to identify patients who would benefit from simultaneous intensification of glycemic control, lipid management, and anti-inflammatory strategies.

AngioPLUS offers specific advantages for IPN assessment in routine care. Unlike OCT and IVUS, which provide microstructural detail but are invasive and resource-intensive, AngioPLUS is noninvasive, contrast-free (23), and integrated into commercially available high-end ultrasound platforms, making it deployable in outpatient and primary care settings where invasive imaging is infeasible (12, 13). Although AngioPLUS detects microflow signals rather than anatomical microvessel contours, these signals have been validated against CEUS as surrogates of plaque inflammatory activity (12, 13). The good interobserver agreement (*κ* = 0.86) observed here, together with the training protocol using 20 pilot images, reinforces the feasibility of standardization. A direct clinical implication is that a brief, structured training program could enable certified sonographers in regional centers to reliably grade IPN, accelerating the decentralization of plaque vulnerability assessment beyond specialized academic hospitals.

### 4.1 Limitations and future research directions

Several considerations should guide future work. First, the semi-quantitative AngioPLUS IPN grading requires validation against CEUS and head-to-head comparison with OCT/IVUS to confirm whether microflow signals reflect microvessel density and fibrous cap infiltration. Second, independent external validation in geographically diverse multicenter cohorts is needed to establish the generalizability of the C-index and time-dependent AUCs. Third, the limited number of recurrence events (*n* = 26, EPV = 13) restricts the exploration of nonlinear relationships and interactions, underscoring the need for larger, event-rich datasets to refine the model and test additional predictors. Fourth, medication adherence was recorded only as a binary variable; future studies should prospectively collect granular data on statin type, dose, and antiplatelet regimens to adjust for treatment intensity. Fifth, operator dependency and signal attenuation in deep plaques may affect grading reproducibility across AngioPLUS-like platforms, warranting a structured multicenter training program with standardized acquisition protocols and central adjudication.

## 5 Conclusion

In summary, the two-variable model incorporating IPN grade 2 and LDL-C provides a practical and biologically grounded tool for stratifying stroke recurrence risk in patients with mild-to-moderate carotid stenosis. With prospective multicenter validation and standardized training, this approach holds substantial promise for guiding individualized, intensity-adapted secondary prevention in diverse clinical settings.

## Data Availability

The raw data supporting the conclusions of this article will be made available by the authors, without undue reservation.
