# Supplementary material for: AngioPLUS-derived carotid intraplaque neovascularization predicts recurrent anterior circulation stroke: a prospective cohort study
Source: Front Neurol. 2026 Aug 11;17:1819400. doi: 10.3389/fneur.2026.1819400 (PMC13503154; doi:10.3389/fneur.2026.1819400)
Supplement: Supplementary file 1 [file Table_1.DOCX]

**Supplementary Table 1**. Comparison of baseline characteristics between the recurrence (n = 26) and non-recurrence (n = 117) groups

| **Variable** | **Non-recurrence group**  **(n =117)** | **Recurrence group**  **(n = 26)** | ***p*-value** |
| --- | --- | --- | --- |
| Age, year, mean ± SD | 67.35±12.17 | 68.54±9.79 | 0.537 |
| Sex, man, n (%) | 74 (63.2) | 18 (69.2) | 0.565 |
| Hypertension, n (%) | 56 (47.9) | 14 (53.8) | 0.581 |
| Diabetes, n (%) | 27 (23.1) | 10 (38.5) | 0.105 |
| Hyperlipidemia, n (%) | 31 (26.5) | 5 (19.2) | 0.440 |
| Coronary heart disease, n (%) | 16 (13.7) | 4 (15.4) | 0.820 |
| History of stroke, n (%) | 28 (23.9) | 9 (34.6) | 0.261 |
| Smoking, n (%) | 39 (33.3) | 10 (38.5) | 0.618 |
| Drinking, n (%) | 33 (28.2) | 8 (30.8) | 0.794 |
| Poor medication adherence, n (%) | 22 (18.8) | 8 (30.8) | 0.175 |
| BMI, mean ± SD | 24.33 ± 2.721 | 25.13 ± 2.283 | 0.099 |
| TG, median (IQR) | 1.44 (1.02, 2.31) | 1.42 (0.85, 4.01) | 0.628 |
| TC, median (IQR) | 4.41 (3.71, 5.02) | 4.45 (4.01, 5.18) | 0.593 |
| HDL-C, mean ± SD | 1.21 ± 0.28 | 1.16 ± 0.266 | 0.902 |
| LDL-C, mean ± SD | 2.75 ± 0.820 | 3.49 ± 0.639 | 0.192 |
| FBG, median (IQR) | 5.28 (4.84, 7.04) | 6.44 (5.43, 9.46) | 0.012 |
| HCY, median (IQR) | 12.00 (10.20, 16.85) | 16.00 (12.02, 20.38) | 0.018 |
| Cr, median (IQR) | 68 (56.00, 81.50) | 67.50 (55.25, 83.75) | 0.969 |
| Thrombolysis, n (%) | 12 (10.3) | 4 (15.4) | 0.453 |
| CIMT | 1.0 (0.8, 1.1) | 1.0 (0.8, 1.1) | 0.906 |
| IPN grade, n (%) |  |  | ＜0.001 |
| Grades 0 and 1, n (%) | 78 (66.7) | 5 (19.2) |  |
| Grades 2, n (%) | 39 (33.3) | 21 (80.8) |  |
| Plaque echogenicity, n (%) |  |  | 0.725 |
| Types I and II | 85 (72.6) | 18 (69.2) |  |
| Types III and IV | 32 (27.4) | 8 (30.8) |  |
| Degree of carotid stenosis |  |  | 0.161 |
| Mild stenosis | 99 (84.6) | 19 (73.1) |  |
| Moderate stenosis | 18 (15.4) | 7 (26.9) |  |
| PSV, mean ± SD | 115.24±11.47 | 115.81±8.97 | 0.813 |

Note: Abbreviations: BMI, body mass index; CIMT, carotid intima-media thickness; Cr, creatinine; FBG, fasting blood glucose; Hcy, homocysteine; HDL-C, high-density lipoprotein cholesterol; IPN, intraplaque neovascularization; IQR, interquartile range; LDL-C, low-density lipoprotein cholesterol; TC, total cholesterol; TG, triglycerides ; PSV, peak systolic velocity;

Patients were stratified into the recurrence (n = 26) and non-recurrence (n = 117) groups based on the incidence of recurrent ischemic stroke.  Baseline characteristics are detailed in Supplementary Table 1.  No significant intergroup differences were observed in demographic parameters.  However, compared with the non-recurrence group, the recurrence group demonstrated significantly higher levels of fasting blood glucose (P = 0.012) and homocysteine (P = 0.018), as well as a higher prevalence of grade 2 IPN (P = 0.002).

**Supplementary Figure 1**. Lasso regression for variable selection.

Plot of the partial likelihood deviance versus log(λ) in the Lasso regression model. The left and right vertical dashed lines indicate the values of lambda.min and lambda.1se, respectively. Two variables (LDL-C and IPN Grades 2) with non-zero coefficients were selected at the optimal lambda.1se value (λ = 0.106).

**Supplementary Figure 2**. Time-dependent concordance index (C-index) for the prediction model validated by Bootstrap resampling.****
The solid line represents the C-index of the model at different follow-up time points, while the dashed horizontal line indicates a C-index of 0.5 (no discriminative ability). The model demonstrated stable predictive performance throughout the follow-up period, with C-index values ranging from 0.71 to 0.90. Most time points exhibited C-index values above 0.77, and the highest value of 0.90 was observed at 54 months. These findings indicate that the model maintained good discriminative ability across all evaluated time points.

**Supplementary Figure 3**. Calibration curves of the nomogram for predicting 12-, 24-, and 36-month recurrence-free survival.

**T**he calibration of the nomogram was assessed using Bootstrap resampling (1,000 replicates). As illustrated in Supplementary Figure 3, the predicted probabilities of recurrence-free survival at 12, 24, and 36 months demonstrated good agreement with the actual observed probabilities estimated by the Kaplan-Meier method. The calibration curves closely approximated the ideal diagonal line across all time points, indicating satisfactory calibration and robustness of the model, with no evidence of significant overfitting or underestimation of risk.
